# Ultra high performance liquid chromatography–high resolution mass spectrometry plasma lipidomics can distinguish between canine breeds despite uncontrolled environmental variability and non-standardized diets

**DOI:** 10.1007/s11306-016-1152-0

**Published:** 2017-01-05

**Authors:** Amanda J. Lloyd, Manfred Beckmann, Thomas Wilson, Kathleen Tailliart, David Allaway, John Draper

**Affiliations:** 1 Institute of Biological Environmental and Rural Sciences, Aberystwyth University, Aberystwyth, UK; 2 WALTHAM Centre for Pet Nutrition, Freeby Lane, Waltham-on-the-Wolds, Melton Mowbray, Leicestershire, UK

**Keywords:** Metabolomics, Lipidomics, Plasma, Canine breeds, Accurate mass metabolomic profiling

## Abstract

**Introduction and objectives:**

The purpose of this study was to use high accurate mass metabolomic profiling to investigate differences within a phenotypically diverse canine population, with breed-related morphological, physiological and behavioural differences. Previously, using a broad metabolite fingerprinting approach, lipids appear to dominate inter- and intra- breed discrimination. The purpose here was to use Ultra High Performance Liquid Chromatography–High Resolution Mass Spectrometry (UHPLC–HRMS) to identify in more detail, inter-breed signatures in plasma lipidomic profiles of home-based, client-owned dogs maintained on different diets and fed according to their owners’ feeding regimens.

**Methods:**

Nine dog breeds were recruited in this study (Beagle, Chihuahua, Cocker Spaniel, Dachshund, Golden Retriever, Greyhound, German Shepherd, Labrador Retriever and Maltese: 7–12 dogs per breed). Metabolite profiling on a MTBE lipid extract of fasted plasma was performed using UHPLC-HRMS.

**Results:**

Multivariate modelling and classification indicated that the main source of lipidome variance was between the three breeds Chihuahua, Dachshund and Greyhound and the other six breeds, however some intra-breed variance was evident in Labrador Retrievers. Metabolites associated with dietary intake impacted on breed-associated variance and following filtering of these signals out of the data-set unique inter-breed lipidome differences for Chihuahua, Golden Retriever and Greyhound were identified.

**Conclusion:**

By using a phenotypically diverse home-based canine population, we were able to show that high accurate mass lipidomics can enable identification of metabolites in the first pass plasma profile, capturing distinct metabolomic variability associated with genetic differences, despite environmental and dietary variability.

**Electronic supplementary material:**

The online version of this article (doi:10.1007/s11306-016-1152-0) contains supplementary material, which is available to authorized users.

## Introduction

The domestic dog displays great levels of morphological, physiological and behavioral diversity (Spady and Ostrander 2008). Previously metabolite fingerprinting, namely Flow Infusion Electrospray MS (FIE-MS) and NMR, have been employed to characterize the main drivers of variance in the plasma and urine metabolomes of dogs (Beckmann et al. 2010; Lloyd et al. 2016; Viant et al. 2007). In controlled environmental conditions with a limited number of breeds the primary drivers have included the individual, the breed and gender (Beckmann et al. 2010; Viant et al. 2007). One of the challenges of working with owner-maintained pets is experimental variability derived from an uncontrolled environment and non-standardized diets. Analysis of urine and plasma metabolomes collected from a larger number of breeds obtained from home-based dogs resulted in less distinct breed discrimination (Beckmann et al. 2010; Lloyd et al. 2016). In a more phenotypically diverse and ‘free-living’ canine population, despite the diet and environmental confounders, some breeds remained distinct from all others (Greyhound, Chihuahua and Dachshund). Furthermore in the two breeds that were present in both the controlled environmental and the client-owned environmental study cohorts (Labrador Retriever and Cocker Spaniel) phosphatidylcholines were identified common to dogs of the same breeds but of different genetic stock and from different environmental conditions (Lloyd et al. 2016). It was speculated that other differences in lipid metabolism and lipid functionality may exist between breeds.

Breed differences in lipids have been reported previously. In a targeted metabolite profiling study investigating individual variability when fed the same diet, metabolites that showed high inter-individual and low intra-individual variance were also related to lipid/sterol chemistry (Colyer et al. 2011). These confirm other non-metabolomics studies: for example total plasma cholesterol and triacylglyceride concentrations have been shown to vary between canine breeds and with diet (Downs et al. 1993; Usui et al. 2014) and Beagles and Greyhounds have been shown to have different pulmonary surfactants (Clercx et al. 1989). Primary hyperlipidemia is usually associated with certain breeds such as Miniature Schnauzers (Xenoulis et al. 2013) and significant differences have been shown between lipoprotein density profiles of different breeds (Downs et al. 1993; Xenoulis et al. 2013), with and without hyperlipidemia (Xenoulis et al. 2013). Additionally, within the same breed of dog, the lipoprotein profile and plasma lipids can be influenced by dietary fat, activity level and lifestyle (Downs et al. 1997). These data from the literature and the consistent finding in our previous studies show that lipids dominate the metabolites that discriminate between breeds and individuals within a phenotypically diverse dog population (Lloyd et al. 2016). In the current study we present more systematic and detailed characterization of the plasma lipidome in dog breeds using Ultra High Performance Liquid Chromatography–High Resolution Mass Spectrometry and suggest a strategy for working with owner-maintained animals in the context of uncontrolled environmental variability and non-standardized diets.

## Materials and methods

### Animal maintenance

The samples are derived from the previously reported study (Lloyd et al. 2016). The client-owned dogs were recruited in Australia (University of New England, UNE) and maintained on different diets and fed according to their owners’ feeding regimen and amounts (see electronic supplementary Table S1 for full diet information). Exclusion criteria were dogs with obvious signs of ill health, pregnant or lactating dogs, bitches in season and dogs considered to be puppy or senior for the breed (veterinarian’s discretion). The breeds represented in the present study were Beagle (Be, n = 12), Chihuahua (Ch, n = 7), Cocker Spaniel (CS, n = 12), Dachshund (Da, n = 8), Golden Retriever (GR, n = 12), Greyhound (Gh, n = 12), German Shepherd (GS, n = 12), Labrador Retriever (LR, n = 12) and Maltese (Ma, n = 9).

### Plasma collection and extraction

A fasted (>12 h fast) blood sample was taken from the jugular vein by trained veterinarians from each individual dog. Blood was collected for both RNA and metabolite fingerprinting analysis. In keeping with reduction and refinement principles of research with animals, the sample volume required was minimised by filtering blood to collect leukocytes for RNA extraction prior to centrifugation of the leukocyte-depleted blood filtrate to obtain plasma. Fasting blood samples (up to 9 ml), collected in EDTA tubes (BD Vacutainer, 10 ml 367,525), were mixed by inversion and passed through a Leukolock filter using a 25G needle into an evacuated plain tube (BD Vacutainer, 10 ml 366,636) on ice. The leukocyte-depleted blood sample was kept on ice until centrifuged (2000 g for 15 min). Plasma samples were collected and stored, either on dry ice for transport or for long-term storage at −80 °C until analysis.

### Lipid extraction of plasma

Plasma was extracted for metabolomic analysis using the following method. Aliquots of chilled plasma (20 μl) on ice were diluted with 10 µl of pre-chilled water (on ice) and 150 µl of pre-chilled methanol (−20 °C), vortexed and then 500 µl MTBE (methyl-tert-butyl ether) was added (Matyash et al. 2008). Samples were then shaken for 60 min at room temperature and 125 µl of water was added before they were centrifuged for 1 min at 14,000 *g* (room temperature). The organic phase (400 µl) was transferred into a new Eppendorf tube and stored at −80 °C until required. Immediately prior to use, the samples were centrifuged for 3 min at 14, 000 *g* (room temperature).

### Ultra high performance liquid chromatography–high resolution mass spectrometry (UHPLC–HRMS) analysis

Extracted plasma samples were analysed on an Exactive Orbitrap (Thermo Fisher Scientific) mass spectrometer, which was coupled to an Accela Ultra High Performance Liquid Chromatography (UHPLC) system (Thermo Fisher Scientific). Chromatographic separation was performed on a reverse phase (RP) Acquity UPLC BEH C_18_ 1.7 µm, 2.1 × 100 mm column (Waters, Milford, USA) using ACN: H_2_O (40:60) with 10 mM ammonium acetate as mobile phase solvent A and ACN: isopropanol (10:90) with 10 mM ammonium acetate as mobile phase solvent B. Each sample (20 μl) was analysed using 30–100% gradient of B in 10 min after washing the column with 100% of B for 2 min and re-equilibrating the column with 70: 30 of A: B for 3.5 min. A flow rate of 400 µl/min was used for running the samples. Column oven temperature was set to 60 °C and the data were acquired in both positive and negative ESI, using a heated electospray ionisation source (HESI). Mass spectra were acquired from 70 to 1400 mass-to-charge ratio (*m*/*z*) using a mass resolution of 100,000. The spray voltage was 4 kV for both ionization modes. The temperature of the ion transfer capillary was 370 °C and sheath and auxiliary gas was 30 and 15 arbitrary units, respectively. The data were recorded using the Xcalibur 2.0.0 software package (Thermo Fisher Scientific). Mass calibration was performed for both ESI polarities before the analysis using a mixture of caffeine, MRFA (L-methionyl-arginyl-phenylalanyl-alanine), Ultramark 1621, sodium dodecyl sulfate (SDS), and sodium taurocholate dissolved in acetonitrile-methanol-water solution with 1% acetic acid.

### UHPLC–HRMS data processing

All data were acquired in profile mode, and converted to centroid mzXML using msconvert (ProteoWizard). All processing was performed using XCMS (Smith et al. 2006) (version 1.3.8) and in R (version 3.0.2). XCMS was used to process raw LC–MS data into a matrix of ~2500 features with corresponding raw intensity values.

### Sample classification and selection of potentially explanatory signals

FIEmspro was used for all multivariate modelling, classification and feature selection (Beckmann et al. 2008; Enot et al. 2008) (URL http://users.aber.ac.uk/jhd/). High accurate mass data was binned to 0.01 amu after raw data processing using bespoke routines, allowing direct comparison of plasma profiles, prior to signal annotation. Principal Component Analysis (PCA) was followed by PC-Linear Discriminant Analysis (PC-LDA). Plots of the first two PC-Discriminant Functions (PC-DFs) allowed visualization of the goodness of class separation as quantified by Tw values (Eigenvalues). Random Forest (RF) was employed in the analysis of the multivariate data and the RF classification ‘margin’, along with the area under the ROC (receiver operating characteristic) curve (AUC) and accuracy (ACC) was used to assess classification performance (Enot et al. 2008). Models were deemed adequate overall if RF margins > 0.2 and AUC and/or ACC values > 0.8, thresholds which we have implemented in previous publications (Enot et al. 2008).

A combination of RF, AUC and student’s *t*-test, were used to highlight potentially explanatory signals responsible for discriminating between sample classes in a full feature rank list (Enot et al. 2008). RF feature selection was performed by calculating Importance Scores (RFIS), being the mean decrease in accuracy over all classes when a feature is omitted from the data. AUC used the area under curve of the sensitivity (true-positive rate) against the specificity (false-positive rate) and student’s *t*-test ranked the features by the absolute value of the P-values.

Randomized re-sampling strategies using bootstrapping were applied in the process of classification and feature selection to counteract the effect of any unknown, structured variance in the data. We used 100 bootstraps in pair-wise comparisons for each of the applied statistical operations with 2/3 of data as training and 1/3 as test set. RF was set to ntree = 1000 for each bootstrap which is adequate considering the dimensionality of data.

Pearson correlation coefficients between selected variables were calculated using the function *cor* in R version 2.5.1 (Enot et al. 2008). Variables with correlation coefficient absolute values (|r|) > 0.8 were considered to belong to a cluster indicative of different ionization or potential biotransformation/breakdown products of a single metabolite.

### Targeted flow infusion electrospray-ionization tandem mass spectrometry and annotation of signals

For metabolite signal annotation, accurate *m*/*z* values were extracted from the un-binned matrix to enable direct identification of metabolites at 1–5 ppm directly in the first pass profile. These were queried using MZedDB, an interactive accurate mass annotation tool which can be used directly to annotate signals by means of neutral loss and/or adduct formation rules (Draper et al. 2009).

Selected ion signals were investigated further using tandem Mass Spectrometry (MS^*n*^) on a Nano-Flow (TriVersaNanoMate, AdvionBioSciences Ltd, UK) LTQ-Fourier Transform-Ion Cyclotron Resonance Ultra-Mass-Spectrometry (FT-ICR-MS; where Ultra refers to the high-sensitivity ICR-cell) as reported previously (Lloyd et al. 2011, 2016). Samples were prepared as for UHPLC-HRMS and reconstituted in methanol/water (80/20, v/v). For each targeted *m*/*z* value a scan window was set for 20 scans, an isolation width of 1 *m*/*z* and using normalized collision energy of 30 V. An activation coefficient ‘Q’ of 0.250 was chosen and an activation time of 30 ms, with wideband activation turned on and a source fragmentation of 20–30 V. Mass range settings were dependent upon the molecular weight of the target ion.

Metabolites were putatively annotated to MSI level 2 without chemical reference standards due to the lack of standard availability, based upon physicochemical properties, retention times and spectral similarity with public/commercial spectral libraries [Lipid Maps, HMDB, Metlin and Massbank (Horai et al. 2010; Sana et al. 2008; Sud et al. 2007; Wishart et al. 2009)]. Choline-containing phospholipid species, Phosphatidylcholine (PCs) and Sphingomyelin (SMs), both show a characteristic *m*/*z* 184 phosphocholine head group peak, as well as an [M + H-59]^+^ peak corresponding to the neutral loss of (CH_3_)_3_N. Whereas fragmentation of phosphatidylethanolamines exclusively yields one peak, an [M + H-141]^+ ^ion from the neutral loss of the phosphoethanolamine head group and fragmentation of phosphatidylserines yields an [M + H-185]^+^ ion from the neutral loss of the phosphatidylserine head group (Pi et al. 2016).

## Results and discussion

### Dog demographics

The full demographics of the study dogs are shown in electronic supplementary Table S1. The mean (±SEM) age of the 96 dogs was 56 months (±3) and had a range of 8–120 months. Using a 9 point body condition score (Laflamme 1997), the mean was 3.0 with a range of 2.0–4.5. The weight varied considerably between breeds (1.8–44.2 kg) with a mean (±SEM) of 20.6 (±0.9 kg). Due to logistical constraints and the focus on the study being breed, recruitment did not result in balanced age and gender within each breed. More female dogs (n = 58) participated in the investigation than males (n = 37), and there was one unrecorded gender; however, there was representation of both females and males in each dog breed class.

### Canine breed clusters can be differentiated in the plasma lipidome

**Fig. 1:**
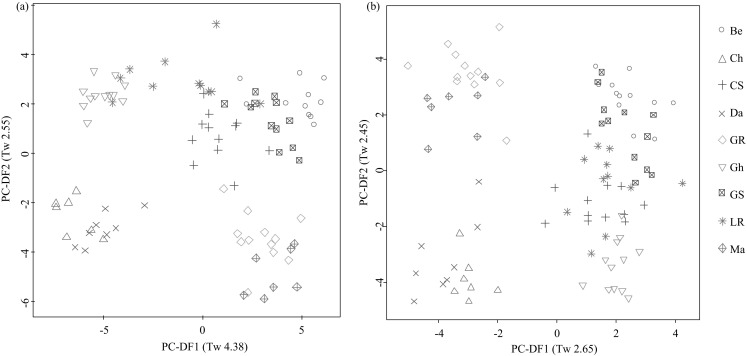
PC-LDA scores plot of positive ionization mode UHPLC-HRMS metabolite profile data representing the fasting plasma metabolome of 9 dog breeds. **a** original data, **b** data with 539 diet signals removed. Where PC-Discriminant Function (PC-DF) Eigenvalues (Tw values) are given in *brackets. Be* beagle, *Ch* chihuahua, *CS* cocker spaniel, *Da* dachshund, *GR* golden retriever, *Gh* greyhound, *GS* german shepherd, *LR* labrador retriever, *Ma* maltese

Previously, we used non-targeted nominal mass fingerprinting to characterize the main drivers of variance in the plasma metabolomes of dogs (Lloyd et al. 2016). The primary fingerprint data consisted of approximately 800 signals at 1 amu in each ionisation mode. In this current work, after pre-processing, data resulted in approximately 2500 signals per profile (with high sensitivity of 1–5 ppm identification level). These data were binned at 0.01 amu for multivariate modelling and classification to allow direct comparison of all plasma profiles. Principal Component-Linear Discriminant Analysis (PC-LDA) indicated that the main source of variance (PC- Discriminant Function 1, PC-DF1, Tw 4.39, Fig. 1a) was between three breeds [Group 1: Ch, Da and Gh (on the negative side of PC-DF1 in Fig. 1a)] and five of the other six breeds (Group 2: Be, CS, GR, GS and Ma; on the positive side of PC-DF1 in Fig. 1a). LR samples were distributed across the first dimension showing intra-breed variation. Separation of breeds into two clusters was also observed in PC-DF2 in which Ch, Da, Ma and GR were distinct from the other five breeds (Tw 2.55, Fig. 1a). The Tw values calculated for the first two dimensions of variance indicated clearly that the plasma lipidome profile of dogs in a fasting state was strongly influenced by breed. As with previous observations using non-targeted metabolome fingerprinting (Lloyd et al. 2016) both breed and breed size appeared significant factors in PC-DF1.

### Identification of the main signals associated with the plasma lipidome of canine breed meta-classes

Prior to investigating possible unique inter-breed plasma lipidome differences, we elected to further characterise the signals causing breed cluster discrimination on PC-DF1 and PC-DF2 seen in Fig. 1a. PCA and PC-LDA models were determined with new meta-classes explaining PC-DF1 and PC-DF2. The first meta-class structure was to explain the variance on PC-DF1 [Group 1 (Ch, Da and Gh) vs Group 2 (Be, CS, GR, GS and Ma)], with LR data omitted due to the lack of distinctive clustering with either of the two groups. The second meta-class designations were structured to elucidate the differences on PC-DF2: Ch, Da, Ma and GR were placed into one meta-class and the other breeds including LR were placed in the comparison meta-class. RF margins, AUC values and ACC modelling scores for these models are shown in electronic supplementary Table S2.

Previously, to characterize the main drivers of variance in the plasma metabolomes of dogs, we used metabolite fingerprinting with targeted high accurate mass analysis, with information acquired on an LTQ-FT-Ultra for metabolite identification, followed by FIE-MS^*n*^ for structual confirmation. In this current work, *m*/*z* data were generated at high resolution and sufficient accuracy to separate signals contained previously in the same 1 amu mass bin, as well as enabling direct identification of metabolites directly in the first pass profile without the need for further targetted accurate mass analysis, only FIE-MS^*n*^ for structual confirmation. Coupling of MS to LC additionally allowed retention times to be associated with every accurate *m*/*z* enabling structural isomers, adducts and isobaric compounds to be distinguished and ionisation adducts to be confirmed.

**Fig. 2:**
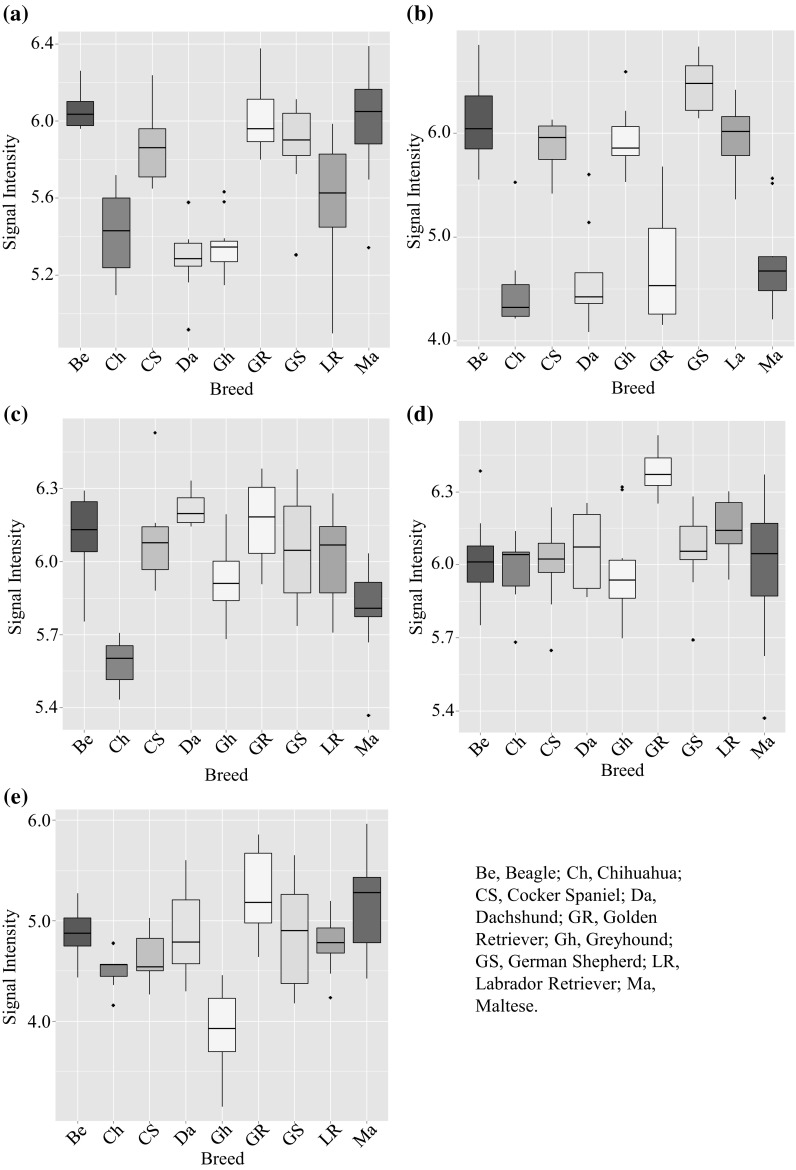
*Box plots* of fasting plasma metabolite signals responsible for dog breed clustering and breed-unique in PC-LDA Figs. 1 and 2. **a** Typical Ch, Da, Gh vs Be, CS, GR, GS, Ma feature (*m*/*z* 720.5525 in Table 1); **b** typical Ch, Da, Ma, GR vs Be, CS, Gh, GS, LR feature (*m*/*z* 268.2990 in Table 2); **c** chihuahua (*m*/*z* 429.3715 in Table 4); **d** golden retriever (*m*/*z* 705.5889 in Table 4); **e** greyhound (*m*/*z* 776.5786 in Table 4). Where the *box* indicates the interquartile range; the *horizontal bar*, the median; *error bars* represent the range of 7–12 dogs. *Be* beagle, *Ch* chihuahua, *CS* cocker spaniel, *Da* dachshund, *GR* golden retriever, *Gh* greyhound, *GS* german shepherd, *LR* labrador retriever, *Ma* maltese

The explanatory signals associated with the discrimination between the meta-classes in Fig. 1a (along PC-DF1 and PC-DF2) were investigated further as the lipidome differences were above adequate thresholds [i.e. RFIS > 0.003 and false discovery rate (FDR) corrected P-values < 0.05; (Enot et al. 2008)]. The top ranked cluster of correlated (|r| > 0.8) signals driving separation on PC-DF1 between Ch, Da and Gh and the rest of the breeds is displayed in Table 1. The remainder of features above the implemented thresholds primarily responsible for the discrimination on PC-DF1 are shown in electronic supplementary Table S2, where correlated signals (|r| >0.8) are highlighted in different colours to show association. These signals were identified as a range of triglycerides, diglycerides and phosphatidylcholines which appeared much reduced in intensity in the Ch, Da and Gh breeds compared with the other breeds (Fig. 2a). Triacylglycerides are the most common and efficient form of stored energy in mammals and higher plasma levels can reflect differences in dietary intake but also hepatic health (Ginsberg 1998; Rifai et al. 1999).

**Table 1:** Annotation of the top correlation cluster explanatory of Chihuahua, Dachshund and Greyhound vs the other breeds (with Labrador Retriever omitted)

UHPLC-HRMS accurate *m*/*z*	RT	RF IS	P-value	Direct annotation using MZedDB*		Theoretical *m*/*z*	Correlation (|r|>0.8)
720.5525	T372	0.0076	0.0001	C_39_H_78_NO_8_P & [M + H]^1+^ = C_39_H_79_NO_8_P	PC (31:0)	720.553784	Cluster 1
748.5836	T398	0.0062	0.0004	C_41_H_82_NO_8_P & [M + H]^1+^ = C_41_H_83_NO_8_P	PC (33:0)	748.585084	Cluster 1
749.5871	T399	0.0052	0.0003	C_41_H_82_NO_8_P & [M + H]^1 + 13^C isotope = C_41_H_83_NO_8_P	PC (33:0)	749.588438	Cluster 1
776.6158	T424	0.0032	0.0017	C_43_H_86_NO_8_P & [M + H]^1+^ = C_43_H_87_NO_8_P	PC (35:0)	776.616384	Cluster 1

*UHPLC–HRMS* ultra high performance liquid chromatography–high resolution mass spectrometry analysis, *RT* retention time, *RF IS* random forest importance score, *PC* phosphatidylcholine, *|r|* correlation coefficient absolute value

*Flow Infusion Electrospray-ionization tandem Mass Spectrometry (FIE-MS^*n*^) was used to confirm PCs due to the characteristic *m*/*z* 184 Phosphatidylcholine head group peak, as well as an [M + H-59]^+^ peak corresponding to the neutral loss of (CH_3_)_3_N

Metabolite identifications driving the separation on PC-DF2 were also assigned using the same strategy (Table 2 and electronic supplementary Table S4). These metabolites appeared reduced in intensity in the Ch, Da, Ma and GR compared with the other breeds (Fig. 2b) and represented different chemical classes to those causing the separation on PC-DF1. Correlation cluster 1 contains the sphingolipid C_17_ sphinganine and long chain hydrocarbons with a single alkene group indicating possible lipid breakdown products. Features in the other two clusters could only be elucidated as unknown sphingomyelins or phospholipids due to the distinctive FIE-MS^*n*^ patterns (electronic supplementary Table S4).

**Table 2:** Annotation of the top correlation cluster explanatory of Chihuahua, Dachshund, Maltese and Golden Retriever vs the other dog breeds

UHPLC-HRMS accurate *m*/*z*	RT	RF IS	P-value	Direct annotation using MZedDB*		Theoretical *m*/*z*	Correlation (|r|>0.8)
268.2990	T159	0.012	<0.0001	C_18_H_34_ & [M + NH_4_]^1+^ = C_18_H_38_N	Unknown	268.299877	Cluster 1
270.3147	T198	0.0119	<0.0001	C_18_H_36_ & [M + NH_4_]^1+^ = C_18_H_40_N	Long chain hydrocarbon with single alkene group	270.315527	Cluster 1
256.2990	T169	0.0108	<0.0001	C_17_H_34_ & [M + NH_4_]^1+^ = C_17_H_38_N	Long chain hydrocarbon with single alkene group	256.299877	Cluster 1
288.2889	T96	0.0094	<0.0001	C_17_H_34_O_2_ & [M + NH_4_]^1+^ = C_17_H_38_NO_2_ or C_1__7_H_3__7_NO_2_ & [M + H]^1+^ = C_17_H_38_NO_2_	Heptadecanoic acid or C_17_ Sphinganine	288.289706	Cluster 1
214.2523	T94	0.0083	<0.0001	C_14_H_28_ & [M + NH_4_] ^1+^ = C_14_H_32_N	Long chain hydrocarbon with a amine group	214.252926	Cluster 1
289.2922	T96	0.0066	0.0010	C_17_H_34_O_2_ & [M + NH_4_]^1+ 13^C = C_17_H_38_NO_2_ or C_17_H_37_NO_2_ & [M + H]^1+ 13^C = C_17_H_38_NO_2_	Heptadecanoic acid or C_17_ Sphinganine ^13^C isotope	289.29306	Cluster 1
244.2627	T68	0.0063	0.0030	C_15_H_30_O & [M + NH_4_]^1+^ = C_15_H_34_NO	Pentadecanal or 9-Pentadecen-1-ol	244.263491	Cluster 1

*UHPLC–HRMS* ultra high performance liquid chromatography–high resolution mass spectrometry analysis, *RT* retention time, *RF IS* random forest importance score, *|r|* correlation coefficient absolute value

*Flow Infusion Electrospray-ionization tandem Mass Spectrometry (FIE-MS^*n*^) was used to confirm annotations due to the distinctive fragmentation patterns

### Diet is one of the main drivers associated with the intra- and inter- variation of plasma lipidomes of different canine breeds

By analysing the meta-data and the habitual eating patterns (see electronic supplementary Table S1) associated with each dog we identified that the discrimination seen in PC-DF1 in Fig. 1a also was impacted by diet types. This is supported by the inter-individual variation seen in LR, as those were distributed in line with whether the dogs followed a diet containing chicken meat (group1; on the negative side of PC-DF1) or red meat (group 2; on the positive side of PC-DF1) as their main protein source. We suggest that the metabolites seen at higher concentrations in group 2 (shown in Table 1 and electronic supplementary Table S3) compared with group 1 correlate with higher red meat consumption by group 2. Raw red meat including beef, lamb and mutton has been shown to contain increased total saturated and monounsaturated fatty acids when compared with white meat such as chicken (Droulez et al. 2006; Williams 2007). Additionally concentrations of total glycerophosphocholines and phosphatidylcholines have also been shown to be much higher in pork/ beef muscle meat and sausages/burgers when compared with chicken muscle meat and processed meat (Zeisel et al. 2003). In human plasma total saturated and monounsaturated fatty acid concentrations increased after a diet high in red meat when compared with a diet high in fatty fish (Wolmarans et al. 1991). Triacylglycerols, derived from glycerol and three fatty acids (both saturated and monounsaturated), have also been shown to increase with high habitual consumption of red meat (Wolmarans et al. 1991) in human plasma. It is important to note that all plasma samples were derived from fasted animals and thus as domesticated dogs may be maintained for many years on the same daily diet it is suggested that any studies on endogenous metabolism should ensure that confounding diet-derived signals are dealt with appropriately.

### Breed cluster differences are enhanced when confounding diet signals are removed from the plasma lipidome of dogs consuming varied diets

We have previously used a data filtering approach to allow the identification of metabolite signals associated specifically with exposure to test foods within a mix of ‘standardized food items’ in a human intervention study (Lloyd et al. 2011). The filtered list included all those explanatory signals with a RFIS > 0.002 after the consumption of the standardised food items when compared with fasting urines. A similar approach was employed here. From the breed model on PC-DF1 (Fig. 1a) the top RFIS ranked signals causing the differences between the binary comparison of the two classes (Ch, Da and Gh vs the other breeds in Table 1), were filtered and removed as they were assumed to be responsible for the confounding diet effect. We incrementally increased the filtering cut-off until the PC-LDA model (Fig. 1a) collapsed and the Ch, Da, Gh vs Be, CS, GR, GS, Ma clustering along PC-DF1 was no longer predominant. This heuristic RFIS threshold was >0.0003; additionally, features which were below the critical threshold, yet showed high correlation with the filtered top ranked signals with a |r| > 0.8, were also omitted from subsequent analyses as these have been shown to be isotopes and adducts in previous analyses. The resultant matrix contained 21.7% fewer features (539 signals out of 2496 were removed) than the complete matrix and when used to model the breed classes the main source of variance (PC-DF1) was now between four breeds (Ch, Da, Ma and GR) and the other five breeds (Tw 2.65, Fig. 1b), as observed previously in PC-DF2 in the original un-filtered model (see Fig. 1a). Notably the LR samples now formed a single distinctive cluster, suggesting the differences in diet were responsible for much of the variance in fasting plasma lipidome. Furthermore, with the exception of GR, the main axis of discrimination (PC-DF1) may be related to breed average weight (Table 1 and electronic supplementary Table S1) as observed previously (Beckmann et al. 2010) in the urine metabolome analysis. The top ranked signals discriminating the Ch, Da, Ma and GR from the other breeds of dogs were identical to those identified previously (Table 2), suggesting shared metabolome similarities in these four breeds. The sphingolipid C_17_ sphinganine and the long chain hydrocarbons with amine groups were lower in the Ch, Da, Ma and GR breeds. Labrador was one of the breeds that had increased concentrations of these discriminatory sphingolipids and related amines when compared with Ch, Da, Ma and GR breeds. Differences in sphingolipid concentrations evident between the breeds in this current study may reflect how the dog breeds differentially transport or mobilise plasma sphingolipids, or may indicate different responses in the insulin signalling pathway between canine breeds (reviewed in Ilan 2016). Also, it has been suggested that endogenous sphinganine may inhibit cholesterol transport in Niemann-Pick Type C (NPC) disease (Roff et al. 1991) providing a suggestion that these dog breeds may have differential cholesterol esterification abilities.

### Unique breed-specific signals are evident when diet-associated signals are filtered from plasma lipidome dataset

One of the objectives of the study was to determine if there were any unique breed-specific signals (i.e. a signal that is not part of a common series of signals discriminating several breeds). Using the diet-filtered dataset, the modelling scores (RF margins, AUC and ACC values) for each breed pair-wise comparison are shown in Table 3. Adequate discrimination modelling scores were shown by CS and LR for only four pair-wise breed comparisons: therefore, we did not pursue these two breeds further (Table 3). For the remaining seven breeds (Ma, GS, Be, Ch, GR, Gh and Da) the modelling scores for ≥6 pair-wise breed comparisons were above the adequate thresholds criteria set, and we sought to identify the explanatory signals specific to each breed. By comparing top ranked signals (RFIS > 0.002 and P-values < 0.05) for each breed comparison, none of the Ma, GS, Da nor Be plasma lipidomes showed any unique breed-specific features consistent across all comparisons. In contrast, consistent unique breed specific features were discovered for the Ch (5 decreased in signal intensity compared to other breeds, example in Fig. 2c), Golden Retriever (7 elevated, example in Fig. 2d) and Greyhound (1 decreased, Fig. 2e). The full RF-IS and P-value data for all pair-wise comparisons are shown in electronic supplementary Table S5. It was interesting to note that despite the absence of any unique breed differences the Beagle plasma lipidome still discriminated from many other breeds suggesting a broad variance in the Beagle plasma lipidome, which is consistent with previous observations in relation to the urinary metabolome of this breed (Beckmann et al. 2010).

**Table 3:** Three classification model ‘robustness’ output statistics Random Forest (RF) margins, area under the receiver-operating characteristic curve (AUC) values and Accuracy (ACC) of UPLC-HRMS data derived from analysis of dog plasma with diet signals filtered out

Breed pair-wise comparison	RF margin	AUC	ACC
Ch vs GR	**0.58**	**1.00**	**0.96**
GR vs Gh	**0.49**	**0.96**	0.87
Be vs Ch	**0.48**	**1.00**	**0.95**
CS vs GR	**0.46**	**0.97**	**0.90**
GR vs Ma	**0.44**	**0.94**	0.89
LR vs Ma	**0.44**	**0.98**	**0.92**
Da vs GR	**0.44**	**0.96**	**0.91**
GR vs GS	**0.42**	**0.95**	0.86
Be vs GR	**0.42**	**0.98**	**0.93**
Ch vs LR	**0.38**	**0.95**	0.85
Ch vs GS	**0.38**	**0.98**	0.87
Be vs Gh	**0.36**	**0.97**	**0.92**
Be vs Ma	**0.36**	**0.94**	**0.90**
Ch vs Gh	**0.35**	**0.96**	0.85
Gh vs Ma	**0.33**	**0.96**	0.85
Ch vs CS	**0.31**	**0.92**	0.78
Be vs Da	**0.30**	**0.92**	0.86
Be vs CS	**0.28**	**0.97**	**0.90**
GR vs LR	**0.28**	**0.93**	0.81
Da vs Gh	**0.27**	**0.94**	0.82
GS vs Ma	**0.25**	**0.91**	0.79
Da vs GS	**0.25**	**0.93**	0.82
Gh vs LR	**0.25**	0.88	0.80
CS vs Da	**0.24**	**0.91**	0.82
Be vs GS	**0.22**	**0.94**	0.81
Da vs Ma	**0.21**	0.89	0.80
CS vs LR	0.19	0.89	0.80
CS vs Ma	0.17	0.78	0.66
GS vs LR	0.17	0.88	0.77
Da vs LR	0.17	0.87	0.76
Gh vs GS	0.14	0.86	0.73
Ch vs Ma	0.14	0.82	0.72
CS vs Gh	0.13	0.86	0.74
Be vs LR	0.12	0.80	0.69
Ch vs Da	0.10	0.80	0.68
CS vs GS	0.09	0.81	0.70

Highlighted in bold are RF margin values > 0.2; AUC > 0.9; ACC > 0.9

*UHPLC–HRMS* ultra high performance liquid chromatography–high resolution mass spectrometry analysis, *Be* beagle, *Ch* chihuahua, *CS* cocker spaniel, *Da* dachshund, *GR* golden retriever, *Gh* greyhound, *GS* german shepherd, *LR* labrador retriever, *Ma* maltese

Direct annotation of signals unique to each of the three breeds, Ch, GR and Gh using MZedDB in the first pass profile was made without the need for further accurate mass analysis (Table 4). We have recently shown that by using a non-targeted ‘global’ metabolomic approach, Ch and Gh were discriminated from other breeds in the study (Lloyd et al. 2016). In Ch the plasma metabolites that were lower than in other breeds appeared to be vitamin D3 sterol lipids and phosphatidylcholines, which is in agreement with a previous report that the size of a dog appears to influence vitamin D3 metabolism (Tryfonidou et al. 2003). It has also been reported that Ch plasma has a low concentration of total cholesterol 197.6 ± 10.07 mg/dL compared with another small dog in this study, the Maltese, (235.2 ± 19.04 mg/dL), as well as larger dog breeds GR (301.1 ± 16.76 mg/dL), LR (229.4 ± 22.45 mg/dL) and CS (228.5 ± 26.34 mg/dL) (Usui et al. 2014). Cholesterol is the main sterol in animal tissues which plays a fundamental role in central metabolic pathways including steroid hormone and vitamin D synthesis (Ginsberg 1998; Rifai et al. 1999). Phosphatidylcholines are predominantly structural lipids important in plasma membranes and lung surfactants as well as being major components in lipoproteins. Inter-breed differences in lipoproteins have been reported previously, with the Cairn Terrier having particularly low levels of Low Density Lipoproteins compared to the LR, Be, Dh and other breeds (Downs et al. 1993).

**Table 4:** Identification of signals explanatory of Chihuahua (Ch), Golden Retriever (GR) and Greyhound (Gh)

Breed	Feature	RT	UHPLC-HRMS accurate *m*/*z*	Direct annotation using MZedDB*			Lipid category
Ch decreased	M429T338	T338	429.3715	C_29_H_48_O_2_	[M + H]^1+^	Hydroxy-dihydro-ethanovitamin D3	Sterol Lipid
	M430T338	T338	430.3792	C_29_H_48_O_2_	[M + H]^1+ 13^C isotope	Hydroxy-dihydro-ethanovitamin D3	Sterol Lipid
	M488T338	T338	488.4324	Unknown			
	M765T360	T360	764.5574	C_44_H_78_NO_7_P	[M + H]^1+^	PC(O-32:6)	Phosphatidylcholine
	M794T367	T367	793.5908	C_46_H_82_NO_7_P	[M + H]^1+ 13^C isotope	PC(O-38:6)	Phosphatidylcholine
GR increased	M589T321	T321	589.4597	C_32_H_61_NO_5_S	[M + NH_4_]^1+^	N-(*trans-* tetradecanoyl)-deoxysphing-4-enine-1-sulphonate	Sphingolipid
	M590T321	T321	590.4631	C_32_H_61_NO_5_S	[M + NH_4_]^1+ 13^C isotope	N-(*trans-* tetradecanoyl)-deoxysphing-4-enine-1-sulphonate	Sphingolipid
	M706T366	T366	705.5889	C_39_H_82_N_2_O_6_P	[M + H]^1+^	SM(d18:0/16:0)	Sphingomyelin
	M707T366	T366	706.5921	C_39_H_82_N_2_O_6_P	[M + H]^1+ 13^C isotope	SM(d18:0/16:0)	Sphingomyelin
	M743T385	T385	742.5728	C_42_H_80_NO_7_P	[M + H]^1+^	PC(O-34:3)	Phosphatidylcholine
	M745T407	T407	744.5887	C_42_H_82_NO_7_P	[M + H]^1+^	PC(O-34:2)	Phosphatidylcholine
	M746T406	T406	745.5921	C_42_H_82_NO_7_P	[M + H]^1+ 13^C isotope	PC(O-34:2)	Phosphatidylcholine
Gh decreased	M777T356	T356	776.5786	C_42_H_83_NO_9_P	[M + H]^1+^	Unknown	

*UHPLC–HRMS* ultra high performance liquid chromatography–high resolution mass spectrometry analysis, *PC* phosphatidylcholine, *SM* sphingomyelin

*Flow Infusion Electrospray-ionization tandem Mass Spectrometry (FIE-MS^*n*^) was used to confirm annotations due to the distinctive fragmentation patterns. Choline-containing phospholipid species, PCs and SMs, both show a characteristic *m*/*z* 184 phosphocholine head group peak, as well as an [M + H-59]^+^ peak corresponding to the neutral loss of (CH_3_)_3_N

The explanatory signal that was at a lower concentration in Greyhound plasma compared with other breeds could not be identified. As Gh generally are seen to have a lower percent body fat than other breeds (Jeusette et al. 2010) and a high percent muscle mass (Drost et al. 2006), it is speculated that this single explanatory signal may be related to the unusual body conformation of the Gh and a robust marker of the Gh breed. The signals elevated in intensity in the Golden Retriever plasma appeared to be sphingolipids, sphingomyelins and phosphatidylcholines. This observation may be consistent with previous evidence of the role of sphingolipids in skin disorders in Golden Retrievers, which are prone to atopic dermatitis (Shaw et al. 2004). Healthy dogs of mixed breeds have been shown to have erythrocytes that contain increased concentrations both of saturated sphingomyelin and also some phosphatidylcholines when compared to dogs suffering with atopic dermatitis (Fuhrmann et al. 2006). Additionally, there is evidence that sphingomyelin undergoes significant interactions with cholesterol (García-Arribas et al. 2016; Ridgway 2000) and Golden Retrievers have been shown to have the highest total cholesterol concentration out of 51 dog breeds studied and a top 14 concentration of total triacylglycerides (Usui et al. 2014).

## Concluding remarks

High accurate mass lipidomic profiling generated data at sufficient accuracy to enable direct identification of metabolites in the first pass profile, without the need for further targeted accurate mass analysis. Within a phenotypically-diverse, home-based canine population, plasma lipidomics data indicated unique breed-specific signals can be discovered and improved when diet-confounding signals were removed. Despite constraints of unbalanced populations for age and gender within the set, robust lipids associated with unique breeds were postulated.

The present data, together with our observations in previous studies (Lloyd et al. 2016), provide further evidence that individuals of closed genetic breed groups can be consistently discriminated from others across different metabolomic platforms and also stress the importance of extensive metadata to evaluate metabolome models. Overall, these data confirm the suggestion that breed development in domesticated dogs has resulted in distinctive, stable metabolic biotypes which, when understood in more depth, may allow improved understanding of healthcare requirements in individual animals.

## Electronic supplementary material

Below is the link to the electronic supplementary material.

Supplementary material 1 (XLSX 1470 KB)

Supplementary material 2 (DOCX 11 KB)
