# Supplementary material for: Ultra high performance liquid chromatography–high resolution mass spectrometry plasma lipidomics can distinguish between canine breeds despite uncontrolled environmental variability and non-standardized diets
Source: Metabolomics. 2017 Jan 5;13(2):15. doi: 10.1007/s11306-016-1152-0 (PMC5216087; doi:10.1007/s11306-016-1152-0)
Supplement: Supplementary file 2 — Supplementary material 2 (DOCX 11 KB) [file 11306_2016_1152_MOESM2_ESM.docx]

**Electronic supplementary material**

S1 Table. Demographics of the 96 dogs included in the study

S2 Table. Three classification model ‘robustness’ output statistics Random Forest (RF) margins, area under the receiver-operating characteristic curve (AUC) values and Accuracy (ACC) of UPLC-HRMS data derived from analysis of dog plasma.

S3 Table. Identification of signals explanatory of Ch, Da and Gh vs the other breeds

S4 Table. Identification of signals explanatory of Ch, Da, Ma and GR vs the other breeds

S5 Table. P-values and Random Forest Importance scores (RFIS) for each breed pair-wise comparison, in retention (RT) time order. Highlighted in grey are the signals causing the discrimination, P- value (adjusted) <0.01 and RF >0.002.
